# Implementing the IMB model combined with rehabilitation nursing to promote rehabilitation and self-management in coronary heart disease patients

**DOI:** 10.3389/fcvm.2026.1864724

**Published:** 2026-08-27

**Authors:** Jinjin Gong, Xinmei Zhang

**Affiliations:** Department of Cardiology, Shandong Provincial Third Hospital, Shandong University, Jinan, China

**Keywords:** combined care, coronary heart disease, IMB model, rehabilitation nursing, rehabilitation-related outcomes, self-management behavior

## Abstract

**Objective:**

To examine the association between routine Information-Motivation-Behavioral Skills (IMB) model-based rehabilitation nursing and rehabilitation-related outcomes and self-management behaviors in patients with coronary heart disease (CHD).

**Methods:**

This single-center retrospective cohort study included 150 patients with CHD admitted to Shandong Provincial Third Hospital from January 2024 to February 2025. After ethics approval, data were extracted from existing clinical and nursing records. Patients were retrospectively classified into a treatment group (documented IMB model combined with rehabilitation nursing, *n* = 75) and a control group (documented conventional care, *n* = 75) according to the nursing care recorded in the source documents. Propensity score matching was used to balance baseline characteristics between the two groups. Repeatedly measured outcomes were analyzed using repeated-measures models, and effect estimates were reported with 95%CIs.

**Results:**

At the 3-month follow-up, the treatment group showed more favorable 6MWD and LVEF than the control group. Cardiac-related parameters, including interventricular septal thickness, left atrial diameter, aortic dilation, and aortic atherosclerosis, were lower in the treatment group. CQQC scores at 1, 2, and 3 months were higher in the treatment group, and repeated-measures analysis indicated a more favorable quality-of-life change pattern over time. The total CSMS score was higher in the treatment group than in the control group (95.86 ± 4.31 vs. 91.14 ± 4.17; *P* < 0.001). Emotional management and disease medical management subscale scores were also higher, whereas the difference in daily management was not statistically significant. Treatment adherence was higher in the treatment group than in the control group (90.67% vs. 69.33%), and SAQ domain scores were also more favorable in the treatment group.

**Conclusion:**

In this retrospective cohort study, the IMB model combined with rehabilitation nursing was associated with more favorable cardiac function, cardiac-related indicators, self-management ability, treatment adherence, and quality-of-life outcomes in patients with CHD. These findings should be interpreted as observational associations and require confirmation in future prospective studies.

## 1 Introduction

Coronary Heart Disease (CHD) is one of the chronic diseases with the highest mortality rates globally, and its disease burden continues to rise (1, 2). According to data from the Guidelines for the Prevention and Treatment of Cardiovascular Diseases in China (2023 Edition), the number of CHD patients in China has exceeded 13 million, with a notable trend toward younger age groups. The incidence rate among individuals under 45 years old has increased by 42% compared to a decade ago. CHD not only leads to acute events such as myocardial ischemia, angina pectoris, and myocardial infarction but also involves a chronic progressive process characterized by cardiac structural remodeling (e.g., left ventricular hypertrophy, left atrial enlargement) and progressive decline in cardiac function (e.g., reduced left ventricular ejection fraction). These processes ultimately result in severe complications such as heart failure and arrhythmias, significantly reducing patients' quality of life and increasing healthcare expenditures (3, 4).

Traditional CHD management models primarily focus on pharmacological treatment and symptom control. However, issues such as patients' insufficient disease awareness, weak self-management abilities, and poor treatment adherence are prevalent. Studies indicate that only 30% of CHD patients consistently adhere to long-term medication regimens, while the implementation rates of non-pharmacological interventions such as exercise rehabilitation, dietary control, and risk factor management are even lower (5–7). This “passive treatment” model leads to a rehospitalization rate as high as 25%–30% and a mortality rate exceeding 50% within five years. Therefore, exploring an integrated nursing model that combines physiological rehabilitation with behavioral interventions has become crucial for improving the long-term prognosis of CHD patients. This study posits that integrating the Information-Motivation-Behavioral Skills (IMB) model with rehabilitation nursing can form a “physiological-psychological-behavioral” tridimensional intervention system. The IMB model provides a theoretical framework, guiding nurses to enhance patients' disease awareness through personalized health education (information), explore patients' intrinsic needs via motivational interviewing (motivation), and strengthen self-management skills through phased rehabilitation training (behavior). Rehabilitation nursing, through specific measures such as exercise prescriptions and respiratory training, directly improves patients' cardiac and physical functions, creating a closed loop of “theory guiding practice and practice feeding back into theory.”

Based on this background, this study retrospectively reviewed the clinical and nursing records of 150 CHD patients admitted between January 2024 and February 2025. Patients were classified into the treatment group or the control group according to whether their source nursing records documented IMB model combined with rehabilitation nursing or conventional care. This study aimed to evaluate whether the IMB model combined with rehabilitation nursing, as documented in routine clinical practice, was associated with better rehabilitation-related outcomes, cardiac-related indicators, and self-management behaviors, thereby providing observational evidence for comprehensive CHD nursing management.

## 2 Objects and methods

### 2.1 Study subjects

This was a single-center retrospective cohort study based on existing clinical and nursing records of patients with CHD admitted to Shandong Provincial Third Hospital from January 2024 to February 2025. After screening according to the predefined inclusion and exclusion criteria, 150 eligible patients were included. No nursing intervention was prospectively assigned, initiated, or monitored by the research team for study purposes. Patients were retrospectively classified according to the nursing care documented in the original clinical and nursing records: patients documented as receiving conventional care were included in the control group, whereas those documented as receiving the IMB model combined with rehabilitation nursing were included in the treatment group. In routine practice, the use of the IMB model combined with rehabilitation nursing was related to the documented nursing care pathway in the cardiovascular department, the availability of nurses trained in this model, and patients' documented willingness and ability to participate in rehabilitation nursing activities after PCI. No random or quasi-random allocation procedure was used. Because the choice of nursing care in routine practice may still have been influenced by clinical workflow, nursing documentation, and patient-related factors, propensity score matching was used to reduce baseline imbalance between the two groups. Details of the propensity score model, matching algorithm, caliper width, and balance diagnostics are described in the statistical analysis section. After matching, 75 patients were retained in each group. In this dataset, all 150 eligible patients were included in the matched analysis, and no eligible patient was excluded during the matching process.

This retrospective cohort study involving human clinical records was reviewed and approved by the Ethics Committee of Shandong Provincial Third Hospital (Approval No. XNLC24-HL03; approval date: 15 September 2024). The study was conducted in accordance with the Declaration of Helsinki and relevant institutional requirements for the protection of medical data. Because all data were obtained retrospectively from existing clinical and nursing records, and no additional intervention, examination, or follow-up was performed for research purposes, the requirement for written informed consent was waived by the Ethics Committee. Before analysis, all patient-identifiable information was removed, and the anonymized dataset was stored in a password-protected database accessible only to authorized research personnel.

### 2.2 Inclusion criteria

The inclusion criteria for this study were developed by comprehensively referencing relevant guidelines, expert consensus, prior research findings, and actual clinical practice at our hospital. All patients were required to have a definitive diagnosis of CHD confirmed by coronary angiography (8, 9) and to be undergoing percutaneous coronary intervention (PCI) for the first time with no more than three stents implanted. Patients had to be adults aged ≤70 years with complete clinical records, a cardiac function classification ranging from Class I to Class III, and the ability to read, understand, and clearly express their wishes. Patients with severe pulmonary diseases or renal insufficiency were excluded, as were those with severe limb dysfunction or neurological disorders. Patients who were clinically unstable after PCI, those who developed other serious diseases affecting cardiac rehabilitation progress or experienced sudden deterioration of CHD, and those who voluntarily withdrew from subsequent cardiac rehabilitation training (rendering completion of the study impossible) were also excluded.

### 2.3 Nursing care documented in clinical records

The following section summarizes the nursing care activities documented in the original clinical and nursing records. These activities were part of routine clinical nursing practice and were not newly assigned or implemented by the research team for the purpose of this retrospective study. Both groups received standard physician-directed post-PCI medical management, medication guidance, routine monitoring, discharge education, and follow-up recommendations according to departmental practice. The main difference between the two groups was the documented nursing care exposure: the control group received conventional care, whereas the treatment group was documented as receiving conventional care plus IMB model combined with rehabilitation nursing. No additional medical treatment was introduced by the research team for study purposes.

The control group was documented as receiving conventional care measures, which included distributing health manuals on CHD and PCI, providing oral explanations about the disease and surgical procedures, answering patients' questions, giving postoperative dietary guidance, monitoring and managing the puncture site, reminding patients to follow prescribed medications, providing general aerobic exercise guidance, and conducting monthly telephone follow-ups after discharge to monitor rehabilitation progress and remind patients to attend follow-up examinations (10, 11).

The treatment group was documented as receiving conventional care plus IMB model combined with rehabilitation nursing, as follows:

Information Intervention: Patients were given a health questionnaire addressing the nursing needs of CHD patients undergoing PCI, covering disease and surgical knowledge, perioperative precautions, and postoperative rehabilitation guidelines. After guiding patients to complete and return the questionnaires, their health needs were systematically organized. Professional knowledge on disease, surgery, and rehabilitation was then conveyed using multimedia tools such as PPT presentations, illustrated brochures, and video explanations. Additionally, a WeChat group for patients was established, inviting patients and their families to join. Relevant knowledge was regularly shared in the group, with scheduled Q&A sessions to encourage learning.
Motivational Intervention: Motivational interviewing was used as the primary communication method to maintain continuous and close interaction with patients. The first interview, conducted on the day after admission, focused on introducing disease mechanisms, risk factors, control strategies, healthy lifestyles, and rational diet planning. Patients' dietary habits were assessed to analyze their motivational states, identify and correct erroneous behaviors and cognitive biases in daily life, and guide them in alleviating negative emotions through appropriate channels. The second interview, held on the day before surgery, evaluated patients' understanding of PCI and self-management, encouraged them to analyze gaps between their behaviors and inner expectations, and explored intrinsic motivations for treatment and self-management to boost confidence. The third interview, conducted on the day before discharge, comprehensively assessed patients' self-management awareness and abilities, identified existing problems, guided patients in analyzing their causes, and jointly explored solutions.

#### 2.3.1 Behavioral skills intervention

The behavioral skills intervention was implemented throughout the patient's hospital stay. Specific measures included guiding patients to maintain regular dietary habits, take medications accurately, and adhere to a consistent sleep schedule; continuously monitoring vital signs and providing detailed explanations of risk factors and preventive measures for adverse events. By assessing patients' self-management awareness and abilities, patients with favorable postoperative prognoses after PCI were invited to return to the hospital and share their self-management experiences, thereby facilitating peer-to-peer knowledge exchange. Personalized training plans were developed based on patients' rehabilitation progress and physical conditions, encouraging participation in activities such as meditation and aerobic exercise. After discharge, regular follow-ups were conducted via telephone or WeChat to monitor patients' daily routines, medication adherence, dietary habits, and exercise behaviors, emphasizing the importance of maintaining a healthy lifestyle. Family members were also engaged to supervise patients in taking medications on time and in the correct dosage, maintaining a balanced diet, and attending regular outpatient follow-ups (12).

#### 2.3.2 Rehabilitation nursing

Differentiated rehabilitation nursing plans were formulated based on patients' cardiac function classifications:

Cardiac Function Class III: During the first 1–2 days after admission, patients performed simple bed-based exercises, including upper limb, neck, and shoulder movements. After symptom alleviation on days 3–4, activities such as stepping and standing beside the bed were gradually introduced, conducted four times daily.
Cardiac Function Class II: During the first 1–2 days after admission, patients engaged in indoor walking training, covering 100–200 meters per session, twice daily. After symptom alleviation on days 3–4, the walking distance was increased to 500 meters per session, with the addition of stair climbing, twice daily.
Cardiac Function Class I: Patients followed a training regimen similar to that of Class II but with increased exercise volume and duration.

All patients were instructed to perform respiratory training, including deep breathing and pursed-lip breathing, for 20 min per session, twice daily. Upon discharge, nursing staff established a WeChat group to distribute rehabilitation training materials, requiring patients to train as instructed, record their progress, and upload records for timely feedback and correction of errors by nursing staff (13).

The documented IMB model combined with rehabilitation nursing was implemented as an integrated nursing care package, including health education, motivational interviewing, behavioral skills training, WeChat-based follow-up, family supervision, peer support, graded exercise training, and respiratory training. This retrospective study evaluated the overall association between this integrated nursing exposure and patient outcomes, rather than the independent effect of each individual component. All patients were followed for a three-month nursing period.

### 2.4 Observation indicators

Rehabilitation-related Outcomes Status was comprehensively evaluated using the six-minute walk test (6MWT) and left ventricular ejection fraction (LVEF). The 6MWT was conducted in a 60-meter hospital corridor with a marked 50-meter segment and markers placed every 3 meters. Patients were instructed to walk back and forth at their fastest pace for 6 min while receiving verbal encouragement, with the total distance walked measured at the end. LVEF was measured and recorded in real time using echocardiography.

Cardiac structure-related parameters were extracted from routine echocardiographic and vascular ultrasound reports at baseline and at the 3-month follow-up, including interventricular septal thickness (mm), left atrial diameter (mm), aortic dilation-related diameter (mm), and aortic atherosclerosis severity index/percentage as recorded in the original imaging report. These reports were generated during routine clinical care by trained ultrasound physicians who were not involved in the nursing care process or the retrospective data analysis. Because group classification was performed retrospectively, imaging assessors were not informed of the study grouping at the time of image interpretation.

Quality of Life was assessed using the Chinese Questionnaire on Quality of Life in Patients with Cardiovascular Disease, which covers six dimensions: physical status, disease control, and psychosocial support. The total score ranges from 0 to 154, with higher scores indicating better quality of life.

Self-Management Ability was evaluated on the day of admission and after three months of intervention using the Coronary Heart Disease Self-Management Behavior Scale developed by Ren Hongyan's team. The scale comprises 27 items across three dimensions (emotional management, disease medical management, and daily management) and uses a five-point Likert scale, with total scores ranging from 27 to 135. Higher scores indicate stronger self-management abilities. The overall reliability coefficient of the scale was 0.913.

Treatment Adherence was assessed by dedicated nurses based on exercise rehabilitation compliance, with the following criteria: full adherence (patients independently completed all training plans as prescribed), partial adherence (patients completed major training components with supervision), and non-adherence (patients failed to adhere to the prescribed training regimen). The adherence rate was calculated as (number of fully adherent patients + number of partially adherent patients)/total number of patients×100%.

Health-Related Quality of Life was evaluated using the Seattle Angina Questionnaire, which includes five domains (angina frequency, disease perception, physical limitation, angina stability, and treatment satisfaction) with 11 items. Each domain is scored out of 100, with higher scores indicating better quality of life. The overall reliability coefficient of the questionnaire was 0.870, with domain-specific reliability coefficients of 0.814, 0.893, 0.825, 0.833, 0.916, 0.857, 0.839, 0.874, 0.902, 0.883, and 0.869, respectively.

### 2.5 Propensity score matching and statistical analysis

SPSS 26.0 was used for data management and statistical analysis, and GraphPad Prism 8 was used for figure preparation. Propensity score matching (PSM) was performed to reduce baseline imbalance between the treatment group and the control group. The propensity score was estimated using a binary logistic regression model, with nursing care group as the dependent variable. Covariates entered into the propensity score model included age, sex, disease duration, cardiac function classification, number of implanted stents, baseline 6-minute walk distance (6MWD), baseline left ventricular ejection fraction (LVEF), and available baseline scale scores. Patients were matched using 1:1 nearest-neighbor matching without replacement, with a caliper width of 0.2 standard deviations of the logit of the propensity score. Covariate balance before and after matching was assessed using standardized mean differences (SMDs), and an SMD <0.10 was considered to indicate acceptable balance. Continuous variables were presented as mean ± standard deviation. Between-group comparisons were performed using independent-samples t tests when normality assumptions were approximately met. For outcomes with available baseline values, analysis of covariance (ANCOVA) was additionally used to adjust for baseline values and relevant covariates. Repeatedly measured outcomes, including CQQC scores at 1, 2, and 3 months, were analyzed using repeated-measures analysis of variance or a linear mixed-effects model with group, time, and group-by-time interaction as fixed effects. Categorical variables were expressed as number and percentage and compared using the chi-square test or Fisher's exact test, as appropriate. Effect estimates were reported with 95% confidence intervals (CIs). For continuous outcomes, mean differences (MDs), 95%CIs, and Cohen's d were calculated. For categorical outcomes, odds ratios (ORs) or risk ratios (RRs) with 95%CIs were calculated. Exact *P* values were reported whenever possible, except when *P* < 0.001. A two-sided *P* < 0.05 was considered statistically significant. Missing data were checked before statistical analysis. Because complete baseline clinical records and follow-up outcome data were required by the inclusion criteria, the final matched dataset contained no missing values for the main variables used in the primary analyses; therefore, no imputation was performed. Given the number of secondary outcomes and subscale comparisons, the Benjamini-Hochberg false discovery rate (FDR) method was used as a sensitivity check for multiple testing. The interpretation of results was based on exact *P* values, FDR-adjusted q values where applicable, effect estimates, 95%CIs, and clinical plausibility.

## 3 Results

After 1:1 nearest-neighbor propensity score matching, 75 patients were retained in each group. In this dataset, all 150 eligible patients were included in the matched analysis, and no eligible patient was excluded during the matching process. The covariates included in the propensity score model were age, sex, disease duration, cardiac function classification, number of implanted stents, baseline 6MWD, baseline LVEF, and available baseline scale scores. After matching, the baseline characteristics were generally balanced between the two groups, with all included covariates showing SMDs <0.10. Detailed covariate balance before and after matching is provided in Supplementary Table S1.

### 3.1 Clinical data

This study included 150 patients after PCI, with 75 in the treatment group and 75 in the control group. Baseline characteristics were well-balanced between the two groups: the treatment group had a mean age of 55.65 ± 7.23 years, with 39 males (52.00%); the control group had a mean age of 55.88 ± 7.71 years, with 38 males (50.67%). Regarding disease duration, the treatment group had 5.01 ± 1.89 years, while the control group had 4.92 ± 1.77 years. No significant differences were observed between the two groups in terms of age, gender, or disease duration (Table 1).

**Table 1:** Clinical data of patients in both groups.

Characteristic	Treatment group	Control group	*^t/χ²^*	*P*
Numbers -	75	75	-	-
Male	39	38	-	-
Gender				
Female	36	37	0.027	0.870
-	45–68	45–68	-	-
Age Mean	55.65 ± 7.23	55.88 ± 7.71	0.189	0.851
Duration of illness Mean	1–8	1–8	-	-
	5.01 ± 1.89	4.92 ± 1.77	0.301	0.764

### 3.2 Rehabilitation-related outcomes

**Figure 1:**
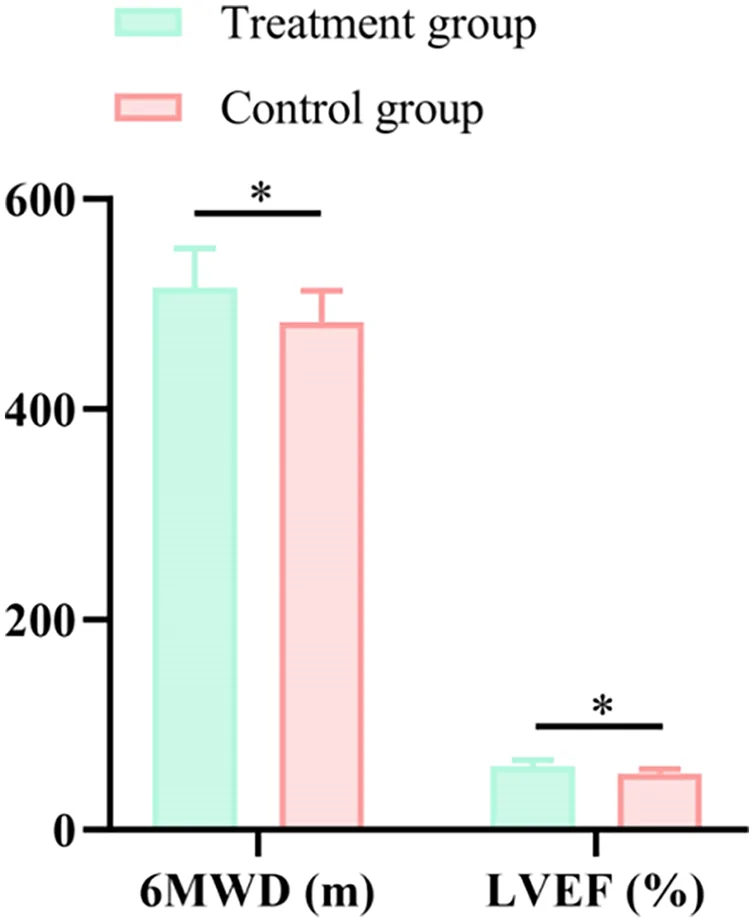
Comparison of rehabilitation-related outcomes indicators between the two groups. Data are presented as mean ± standard deviation or percentage, as appropriate. Exact *P* values, effect estimates, 95%CIs, and effect sizes are reported in the corresponding Results section.

At the 3-month follow-up, the treatment group demonstrated a longer 6-minute walk distance (6MWD) than the control group (515.56 ± 37.56 m vs. 483.14 ± 29.73 m; MD = 32.42 m, 95%CI: 21.48–43.36; Cohen's d = 0.96; *P* < 0.001). LVEF was also higher in the treatment group than in the control group (60.71 ± 5.72% vs. 53.89 ± 3.68%; MD = 6.82%, 95%CI: 5.27–8.37; Cohen's d = 1.42; *P* < 0.001) (Figure 1).

### 3.3 Cardiac structure-related parameters

**Figure 2:**
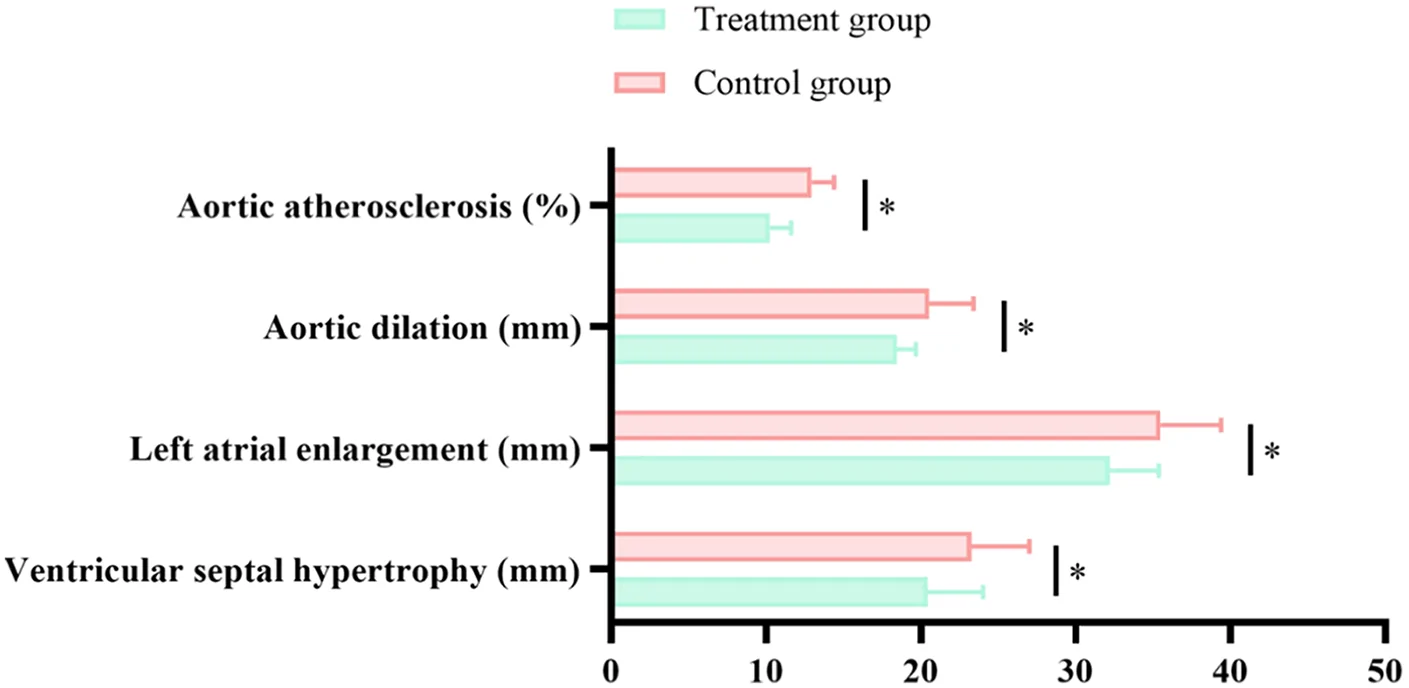
Comparison of cardiac structure-related parameters between the two groups. Data are presented as mean ± standard deviation or percentage, as appropriate. Exact *P* values, effect estimates, 95%CIs, and effect sizes are reported in the corresponding Results section.

Baseline cardiac structure-related parameters were comparable between the two groups after propensity score matching, as shown in Supplementary Table S2. At the 3-month follow-up, the treatment group had lower recorded values of cardiac structure-related parameters than the control group. Specifically, interventricular septal thickness was lower in the treatment group (20.44 ± 3.56 mm vs. 23.26 ± 3.74 mm; MD = −2.82 mm, 95%CI: −4.00–−1.64; Cohen's d = −0.77; *P* < 0.001), as was left atrial diameter (32.19 ± 3.19 mm vs. 35.47 ± 3.92 mm; MD = −3.28 mm, 95%CI: −4.43–−2.13; Cohen's d = −0.92; *P* < 0.001). Aortic dilation-related diameter was also lower in the treatment group (18.47 ± 1.22 mm vs. 20.56 ± 2.84 mm; MD = −2.09 mm, 95%CI: −2.80–−1.38; Cohen's d = −0.96; *P* < 0.001), as was the aortic atherosclerosis severity index/percentage recorded in the original imaging report (10.27 ± 1.35% vs. 12.97 ± 1.43%; MD = −2.70%, 95%CI: −3.15–−2.25; Cohen's d = −1.94; *P* < 0.001) (Figure 2).

### 3.4 Quality of life

**Figure 3:**
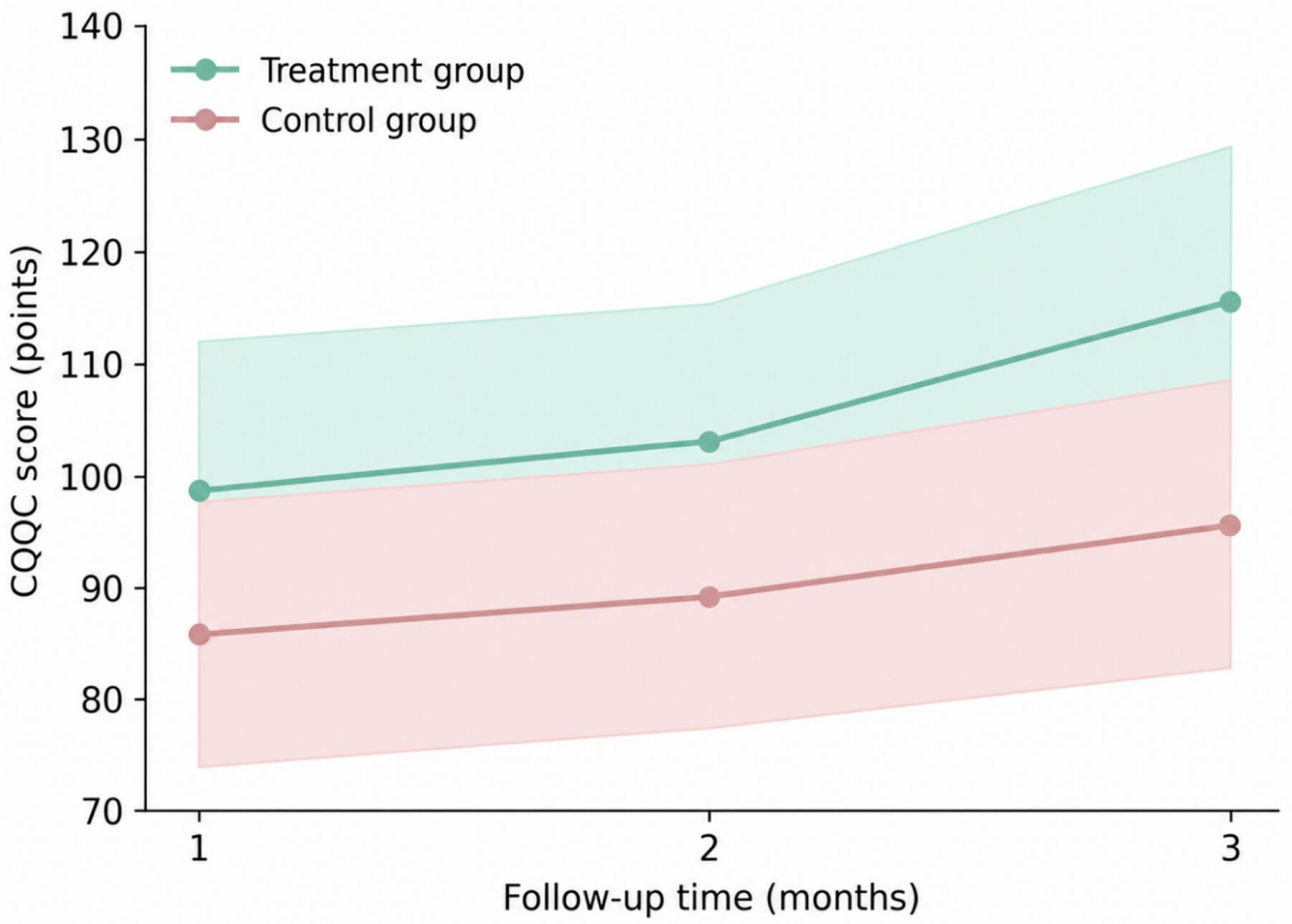
Comparison ofCQQC scores between the two groups. CQQC scores at 1, 2, and 3 months were analyzed using a repeated-measures model with group, time, and group-by-time interaction. Data are presented as mean ± standard deviation.

Repeated-measures analysis showed significant effects of time (F_time = 47.86, *P* < 0.001), group (F_grou*p* = 55.37, *P* < 0.001), and the group-by-time interaction (F_interaction = 9.84, *P* < 0.001), indicating that the pattern of quality-of-life change differed between the two groups over the 3-month follow-up period. In *post hoc* comparisons, the treatment group had higher CQQC scores than the control group at 1 month (98.62 ± 12.31 vs. 85.76 ± 11.83; MD = 12.86, 95%CI: 8.96–16.76; Cohen's d = 1.07; *P* < 0.001), 2 months (102.83 ± 11.33 vs. 89.11 ± 11.77; MD = 13.72, 95%CI: 9.99–17.45; Cohen's d = 1.19; *P* < 0.001), and 3 months (115.24 ± 13.52 vs. 95.47 ± 12.74; MD = 19.77, 95%CI: 15.53–24.01; Cohen's d = 1.51; *P* < 0.001) (Figure 3).

### 3.5 Self-Management

**Figure 4:**
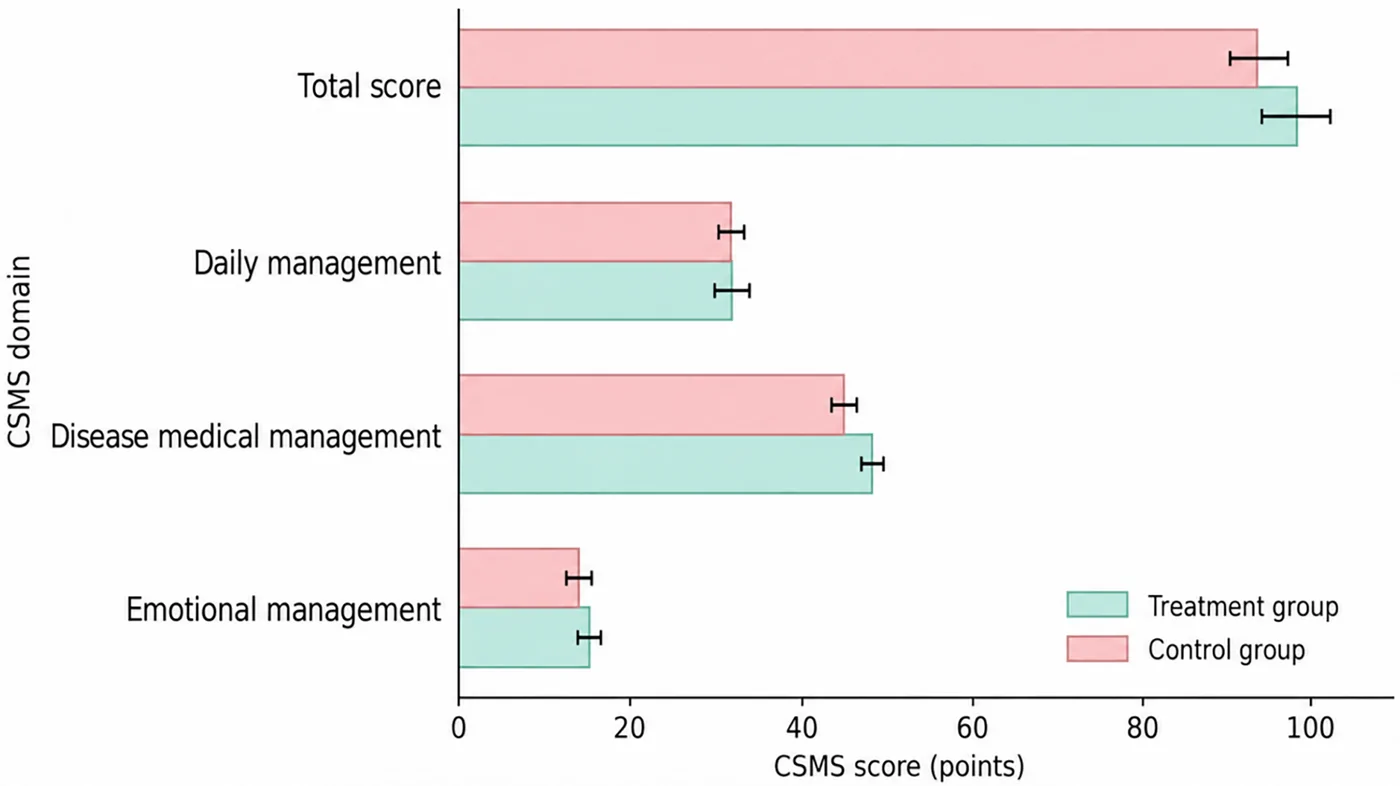
Comparison of CSMS subscale scores and total scores between the two groups. Data are presented as mean ± standard deviation or percentage, as appropriate. Exact *P* values, effect estimates, 95%CIs, and effect sizes are reported in the corresponding Results section.

At the 3-month follow-up, the treatment group had a higher total CSMS score than the control group (95.86 ± 4.31 vs. 91.14 ± 4.17; MD = 4.72, 95% CI: 3.35–6.09; Cohen's d = 1.11; *P* < 0.001). For the CSMS subscales, emotional management (15.24 ± 1.23 vs. 14.11 ± 1.45; MD = 1.13, 95% CI: 0.70–1.56; Cohen's d = 0.84; *P* < 0.001) and disease medical management (49.06 ± 1.17 vs. 45.58 ± 1.44; MD = 3.48, 95% CI: 3.06–3.90; Cohen's d = 2.65; *P* < 0.001) scores were higher in the treatment group. The daily management subscale was numerically higher in the treatment group, but the between-group difference was not statistically significant (31.56 ± 1.91 vs. 31.45 ± 1.28; MD = 0.11, 95% CI: −0.42–0.64; Cohen's d = 0.07; *P* = 0.679) (Figure 4).

### 3.6 Treatment adherence

**Figure 5:**
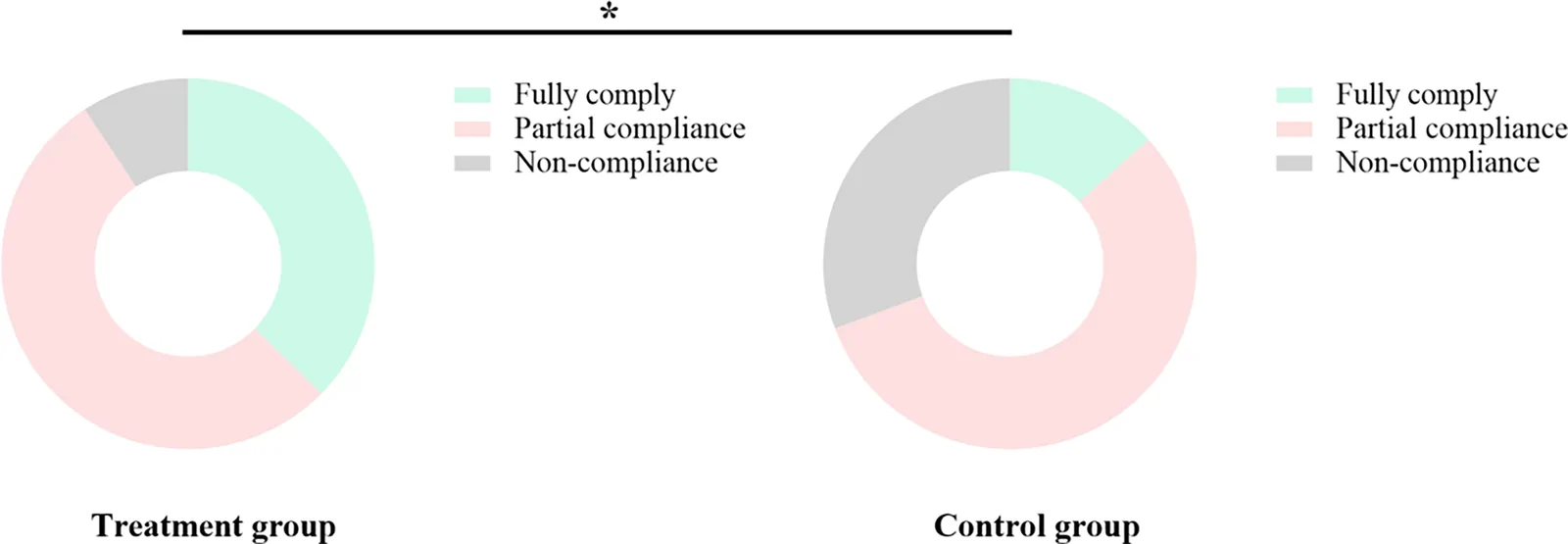
Comparison of treatment adherence between the two groups. Data are presented as mean ± standard deviation or percentage, as appropriate. Exact *P* values, effect estimates, 95%CIs, and effect sizes are reported in the corresponding Results section.

Treatment adherence was higher in the treatment group than in the control group (68/75, 90.67% vs. 52/75, 69.33%; *χ*² = 10.67; *P* = 0.001). The treatment group had a higher likelihood of adherence than the control group (OR = 4.30, 95%CI: 1.71–10.78; RR = 1.31, 95%CI: 1.11–1.55) (Figure 5).

### 3.7 Health-related quality of life

**Figure 6:**
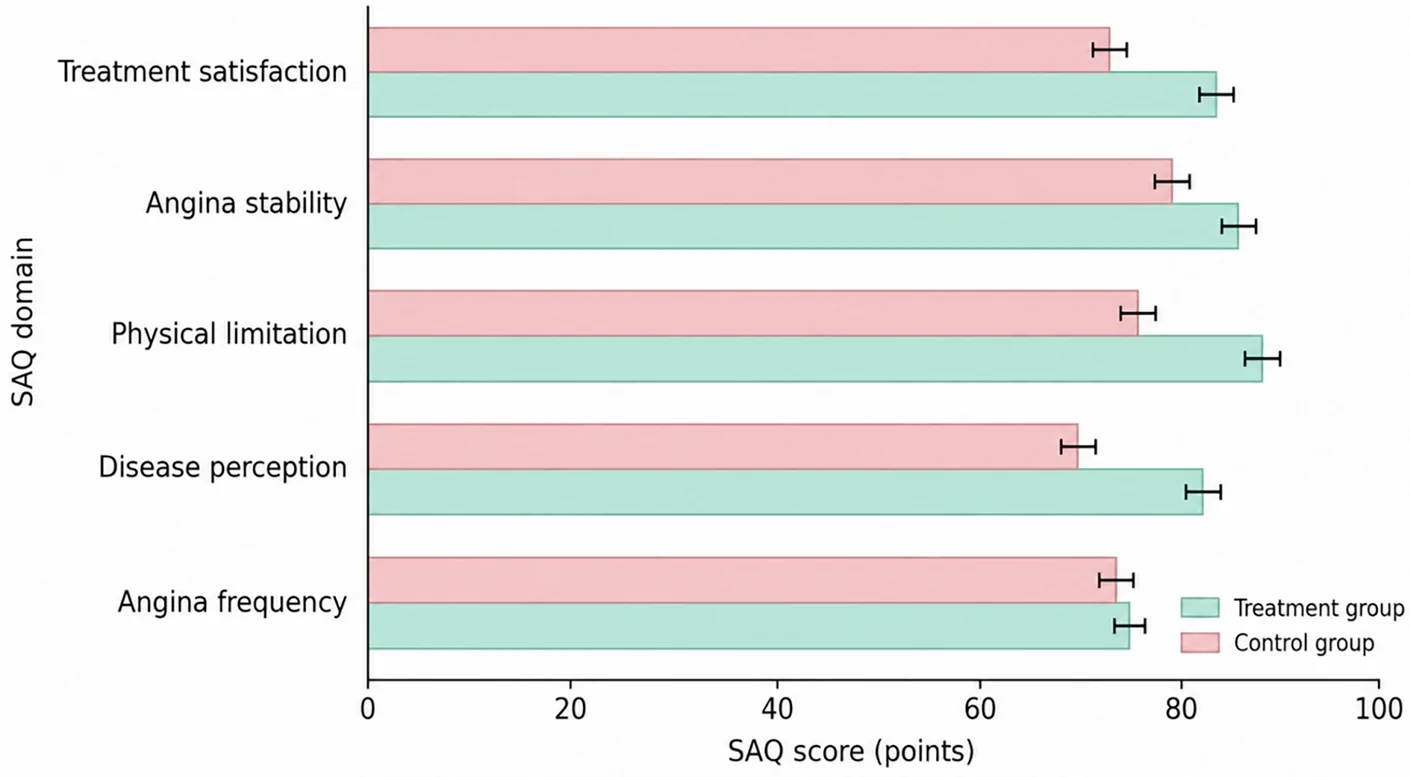
Comparison of SAQ subscale scores between the two groups. *Indicates a significant difference between the two groups, *P* < 0.05.

At the 3-month follow-up, all SAQ subscale scores were higher in the treatment group than in the control group. The mean differences were 8.66 for angina frequency/severity (95%CI: 8.13–9.19; Cohen's d = 5.28; *P* < 0.001), 11.91 for disease perception (95%CI: 11.42–12.40; Cohen's d = 7.87; *P* < 0.001), 10.65 for physical limitation (95%CI: 10.10–11.20; Cohen's d = 6.21; *P* < 0.001), 5.72 for angina stability (95%CI: 5.27–6.17; Cohen's d = 4.08; *P* < 0.001), and 11.12 for treatment satisfaction (95%CI: 10.69–11.55; Cohen's d = 8.39; *P* < 0.001) (Figure 6). After Benjamini–Hochberg FDR correction, all findings that were significant in the primary analyses remained statistically significant (all adjusted q < 0.05), whereas the between-group difference in the daily management subscale remained non-significant.

## 4 Discussion

The Information-Motivation-Behavioral Skills Model (IMB), proposed by Fisher and colleagues in 1992, is a comprehensive intervention framework based on behavioral change theory. This model emphasizes the synergistic effects of information dissemination (e.g., disease knowledge, rehabilitation skills), motivation enhancement (e.g., fostering intrinsic drive, leveraging social support networks), and behavioral skills training (e.g., time management, goal setting) to promote the sustainability of healthy behaviors (14, 15). In recent years, the IMB model has demonstrated significant advantages in chronic disease management: a randomized controlled trial (RCT) targeting patients with type 2 diabetes revealed that IMB-based interventions improved glycemic control compliance rates by 28% and self-management behavior scores by 35%; another study applying the IMB model combined with home follow-up in hypertensive patients showed a 12 mmHg reduction in systolic blood pressure, with effects persisting for 12 months post-intervention. Rehabilitation nursing, as an independent discipline, aims to improve cardiopulmonary function, alleviate anxiety and depression, and enhance activities of daily living through systematic training (e.g., exercise therapy, respiratory training) and psychological support. Research confirms that regular cardiac rehabilitation can reduce all-cause mortality by 27% and rehospitalization risk by 31% in patients with coronary heart disease (16). However, traditional rehabilitation nursing often focuses primarily on physiological function recovery, neglecting the underlying mechanisms of behavioral change (e.g., motivation activation, skill internalization), which limits the long-term maintenance of intervention effects (17). This retrospective cohort study compared outcomes between the treatment group and the control group according to nursing care documented in routine clinical and nursing records. The treatment group showed more favorable cardiac function indicators, cardiac-related parameters, quality of life scores, self-management ability, and treatment adherence than the control group. However, these findings should be interpreted as observational associations rather than definitive causal effects. Although propensity score matching was used to reduce baseline imbalance, residual confounding, selection bias, information bias from medical records, self-report bias from scale-based outcomes, and non-blinding cannot be fully excluded. In addition, the treatment group was documented as receiving a multicomponent nursing package, and the observed outcome differences cannot be attributed to any single element, such as health education, motivational interviewing, family supervision, WeChat follow-up, peer support, exercise training, or respiratory training alone. Several possible explanations may account for these findings. First, the treatment group was documented as receiving a more structured rehabilitation nursing program based on cardiac function classification. Compared with conventional care, this approach provided more individualized exercise guidance and follow-up support. Patients with different cardiac function classifications received graded rehabilitation guidance, ranging from simple bed-based activities to indoor walking, stair-climbing training, and increased exercise duration according to tolerance. This progressive and stepwise training approach helped ensure exercise safety while promoting cardiopulmonary adaptation through sustained rehabilitation stimuli (18–20). In addition, the treatment group was documented as receiving respiratory training and behavioral skills support, including deep breathing, pursed-lip breathing, dietary guidance, medication reminders, emotional regulation, and follow-up feedback. These components may have helped patients better understand rehabilitation goals and translate health education into daily self-management behaviors. Therefore, the more favorable 6MWD and LVEF observed in the treatment group may be related to structured rehabilitation guidance, behavioral skills training, and continued post-discharge support. However, given the retrospective cohort design, these explanations should be interpreted as plausible associations rather than confirmed causal mechanisms.

The cardiac structure-related findings should be interpreted cautiously. After verification of the original imaging records, these indicators were derived from routine echocardiographic or vascular ultrasound reports, and baseline values were comparable between the two groups after propensity score matching. At the 3-month follow-up, the treatment group had lower recorded values of interventricular septal thickness, left atrial diameter, aortic dilation-related diameter, and aortic atherosclerosis severity index/percentage than the control group. These differences may be related to better short-term rehabilitation participation, medication adherence, hemodynamic stability, and risk-factor management among patients documented as receiving the integrated nursing care package. However, cardiac remodeling and atherosclerotic changes usually evolve over a longer period. Therefore, the present retrospective data cannot confirm that nursing exposure directly reversed cardiac structural remodeling or atherosclerosis, and these imaging-recorded differences require confirmation in future prospective studies with standardized imaging protocols and longer follow-up.

The improvement in quality of life in the treatment group exhibited a time-dependent pattern, with CQQC scores consistently higher than those in the control group from 1 month to 3 months. Due to limited follow-up frequency and relatively uniform educational content, conventional care may not fully meet patients' evolving rehabilitation needs after discharge. In contrast, the treatment group was documented as having WeChat-based follow-up, regular educational content sharing, rehabilitation feedback, and communication with nurses and peers, which may have provided more continuous post-discharge support. This digital follow-up approach may have helped patients obtain timely disease-management information, share rehabilitation experiences, and maintain engagement during the rehabilitation process. Recent studies on telemedicine and technology-assisted rehabilitation have also emphasized that digital support should not be viewed only as a technical tool, but as a way to improve patient experience, continuity of care, engagement, self-management, and adherence after discharge (21–23). At the same time, the social support provided through nurse-patient communication, peer interaction, and family involvement may have helped alleviate patients' feelings of loneliness and anxiety and enhance self-efficacy, which is consistent with previous studies on psychological support and behavioral factors in patients with cardiovascular disease (24–26). Meanwhile, the three structured motivational interviews conducted during the motivation intervention phase—at admission, the day before surgery, and the day before discharge—formed a closed-loop management process of “assessment-feedback-adjustment” by exploring patients' intrinsic motivations, analyzing behavioral cognitive biases, and jointly developing solutions. These elements may have provided continuous psychological support and behavioral guidance, thereby contributing to more favorable quality-of-life scores. However, because WeChat follow-up, peer support, motivational interviewing, and rehabilitation guidance were delivered as part of an integrated nursing care package, the contribution of each individual component cannot be determined separately in this retrospective study.

The improvement in self-management ability in the treatment group was mainly reflected in the higher total CSMS score and higher emotional management and disease medical management subscale scores. However, the daily management subscale did not differ significantly between the two groups after recalculation. Conventional care often failed to help patients translate health knowledge into practical actions due to the absence of systematic behavioral skills training. The treatment group developed personalized training protocols and guided meditation practices during the behavioral skills intervention phase, enabling patients to master specific disease management skills (e.g., regular medication use, balanced diet) while learning psychological adjustment techniques such as mindfulness meditation to alleviate negative emotions. The information intervention phase employed diverse formats, including PowerPoint presentations, brochures, and videos, to deliver professional knowledge, facilitating a shift from “passive receipt of information” to “active learning” through questionnaire feedback and health needs assessment. The motivation intervention phase continuously reinforced patients' intrinsic drive through three structured interviews, fostering greater autonomy and persistence across emotional management, disease medical management, and daily management dimensions. This three-dimensional improvement in “knowledge-motivation-skills” collectively contributed to an improvement in self-management ability (27–29).

The marked increase in treatment adherence in the treatment group was demonstrated by a substantial rise in adherence rates. Conventional care often led to relaxed self-management among patients as symptoms improved, due to the lack of effective supervision and incentive mechanisms. The treatment group established a tripartite supervision network involving “hospitals-families-patients” by leveraging family supervision and strengthening regular follow-up. Family members provided direct oversight of medication and diet while enhancing patients' treatment confidence through emotional support. Additionally, post-discharge follow-up via phone or WeChat enabled nurses to promptly identify behavioral changes and offer targeted guidance, with this sustained attention and feedback mechanism effectively reducing behavioral deviations and improving overall adherence (30–32).

The improvement in health-related quality of life in the treatment group was reflected by more favorable SAQ domain scores, including angina frequency, disease perception, physical limitation, angina stability, and treatment satisfaction. Conventional care often disrupted patients' daily lives due to recurrent angina attacks, stemming from the absence of systematic angina management strategies. The treatment group reduced both the frequency and severity of angina attacks through exercise training and behavioral skills interventions during rehabilitation nursing. Exercise training lowered the angina threshold by improving myocardial perfusion and enhancing collateral circulation, while behavioral skills interventions minimized triggering factors through regular medication use and dietary control (33, 34). Meanwhile, enhanced disease awareness enabled patients to more accurately identify angina symptoms and adopt appropriate coping measures, while reduced physical limitations allowed greater freedom in daily activities. The stabilization of angina symptoms and improved treatment satisfaction collectively represented the comprehensive enhancement of health-related quality of life.

## 5 Limitations

This study has several limitations. First, this was a single-center retrospective cohort study, and nursing exposure was identified from existing clinical and nursing records rather than prospectively assigned by the research team. Although propensity score matching was used to balance baseline characteristics between the two groups, residual confounding, selection bias, and information bias cannot be fully excluded. In routine clinical practice, the reasons why patients received different nursing care approaches may have been influenced by clinical workflow, ward practice, nursing documentation, patient preference, or other unmeasured factors. Therefore, the present findings should be interpreted as observational associations rather than definitive causal evidence.

Second, all participants were recruited from the cardiovascular department of a single hospital, which may limit the generalizability of the findings to CHD patients from other regions, institutions, or healthcare settings. Future multicenter studies with larger sample sizes are needed to further verify the applicability of these findings. In addition, the follow-up period was limited to 3 months. Although short-term improvements in rehabilitation-related indicators, cardiac function, quality of life, self-management ability, and adherence were observed, this study could not determine whether these changes would persist over a longer period or translate into improved long-term outcomes, such as reduced readmission rates or fewer cardiovascular events.

Third, the IMB model combined with rehabilitation nursing included multiple components, such as information dissemination, motivational interviewing, behavioral skills training, WeChat-based follow-up, family supervision, peer support, follow-up support, and rehabilitation training. Because these components were delivered as an integrated nursing care package in routine practice, the independent contribution of each component could not be determined. In addition, although both groups received standard physician-directed post-PCI medical management and routine discharge guidance, unmeasured differences in co-interventions, nursing attention, follow-up intensity, nurses' communication skills, implementation fidelity, documentation quality, and patient cooperation may have influenced the observed outcomes. Therefore, performance bias and implementation heterogeneity cannot be fully excluded.

Fourth, some outcome measures were based on medical record documentation and patient self-reported scales, including CQQC, CSMS, and SAQ. Medical records may contain incomplete or inconsistently recorded information, and self-reported outcomes may be affected by recall bias or social desirability bias. Although these scales are commonly used in clinical assessment, further validation in different CHD populations may still be needed. Future studies should combine subjective scales with more objective indicators, such as cardiopulmonary exercise testing, biomarker monitoring, and standardized echocardiographic assessment with blinded review where feasible, to improve the reliability and clinical interpretability of outcome evaluation.

Finally, the control group was documented as receiving conventional care, but variations may have existed in the implementation of conventional nursing practice, such as follow-up frequency, depth of health education, and rehabilitation guidance. Such operational heterogeneity may affect the rigor of between-group comparisons. Future prospective studies should adopt clearer intervention protocols, standardized quality-control procedures, longer follow-up periods, and preferably multicenter designs to further confirm the clinical value of the IMB model combined with rehabilitation nursing in patients with CHD.

## 6 Conclusion

In summary, in this retrospective cohort study, the treatment group showed more favorable rehabilitation-related outcomes, cardiac-related indicators, self-management ability, treatment adherence, and quality of life than the control group. Because of the retrospective design, these findings should be interpreted as observational associations rather than definitive causal effects. Future multicenter prospective studies are needed to further confirm the clinical value of the IMB model combined with rehabilitation nursing in patients with CHD.

## Data Availability

The original contributions presented in the study are included in the article/Supplementary Material, further inquiries can be directed to the corresponding author/s.
