# Supplementary material for: Implementing the IMB model combined with rehabilitation nursing to promote rehabilitation and self-management in coronary heart disease patients
Source: Front Cardiovasc Med. 2026 Aug 27;13:1864724. doi: 10.3389/fcvm.2026.1864724 (PMC13579620; doi:10.3389/fcvm.2026.1864724)
Supplement: Supplementary file 1 [file Table1.docx]

Supplementary Table S1

| Covariate | SMD after matching |
| --- | --- |
| Age | 0.031 |
| Sex | 0.027 |
| Disease duration | 0.049 |
| Cardiac function classification | 0.064 |
| Number of implanted stents | 0.053 |
| Baseline 6MWD | 0.082 |
| Baseline LVEF | 0.067 |
| Baseline CQQC score | 0.078 |
| Baseline CSMS score | 0.074 |
