# Supplementary material for: Implementing the IMB model combined with rehabilitation nursing to promote rehabilitation and self-management in coronary heart disease patients
Source: Front Cardiovasc Med. 2026 Aug 27;13:1864724. doi: 10.3389/fcvm.2026.1864724 (PMC13579620; doi:10.3389/fcvm.2026.1864724)
Supplement: Supplementary file 2 [file Table2.docx]

Supplementary Table S2. Baseline and 3-Month Cardiac Structure-Related Parameters

| Outcome | Baseline treatment group | Baseline control group | SMD after matching | 3-month treatment group | 3-month control group | MD | 95%CI | P value | FDR q |
| --- | --- | --- | --- | --- | --- | --- | --- | --- | --- |
| Interventricular septal thickness, mm | 23.51±3.68 | 23.44±3.71 | 0.019 | 20.44±3.56 | 23.26±3.74 | −2.82 | −4.00 to −1.64 | <0.001 | <0.001 |
| Left atrial diameter, mm | 35.76±3.55 | 35.69±3.61 | 0.020 | 32.19±3.19 | 35.47±3.92 | −3.28 | −4.43 to −2.13 | <0.001 | <0.001 |
| Aortic dilation-related diameter, mm | 20.81±2.39 | 20.74±2.45 | 0.029 | 18.47±1.22 | 20.56±2.84 | −2.09 | −2.80 to −1.38 | <0.001 | <0.001 |
| Aortic atherosclerosis severity index, % | 13.09±1.48 | 13.05±1.51 | 0.027 | 10.27±1.35 | 12.97±1.43 | −2.70 | −3.15 to −2.25 | <0.001 | <0.001 |
